# Data analysis of the mortality of cattle and sheep recorded in a sample of Australian saleyards

**DOI:** 10.1017/awf.2025.10058

**Published:** 2026-01-15

**Authors:** Barbara Padalino, Naod Thomas Masebo, Maria Gaia Angeloni, Clive Julian Christie Phillips

**Affiliations:** 1 Department of Agricultural and Food Sciences, University of Bologna, 40127 Bologna, Italy; 2 Faculty of Science and Engineering, Southern Cross University, Lismore, NSW, Australia; 3 Curtin University Sustainability Policy Institute, Kent St., Bentley, Western Australia 6102, Australia; 4 Institute of Veterinary Medicine and Animal Sciences, Estonian University of Life Sciences, Kreutzwaldi 1, 51006 Tartu, Estonia

**Keywords:** Animal welfare, death, fitness, risk factors, trade, transport

## Abstract

In Australia, nearly twenty million cattle and sheep pass through saleyards annually, with potentially significant impacts on their welfare. This study documented the mortality rate occurring from January 2021 to December 2024 at a sample of saleyards of cattle and sheep in New South Wales, Australia, and identified possible risk factors. A database of the number of animals sold and deceased, either on arrival or while contained at each saleyard on each sale day, was created from the National Livestock Identification System. Descriptive statistics, and uni- and multivariable linear regression were used to examine risk factors for mortality. The mean sale mortality rates were 0.016 and 0.096% for cattle and sheep, respectively. In the univariate model, cattle sale mortality rate was associated with the maximum daily temperature, year, size of saleyard, and saleyard location, while minimum daily temperature, region, and saleyard location were associated with sale mortality of sheep. In the multivariable model, size of saleyard, saleyard location, month and year were significant predictors for the cattle mortality rate, while saleyard location and minimum daily temperature remained significant predictors of sheep mortality rate. Furthermore, sale mortality rate was eight times higher in sheep than in cattle, and sheep mortality was higher than values reported in the literature for farms. Further studies investigating the cause of death, journey conditions, and management practices of saleyards are recommended.

## Introduction

Australia has approximately 94 million head of livestock, and a human population of 26.3 million inhabitants (FAOSTAT 2025a,b). Therefore, most ruminant products are exported, especially meat, wool, and animals for breeding (Harper *et al.* 2019). Australia produces around 4% of the world’s beef, accounts for about 1.5% of the world’s cattle inventory, and is the first and fourth largest exporter of sheep meat and beef, respectively (Meat and Livestock Australia 2022a, 2024). In particular, in New South Wales (NSW), there were around 4.4 million head of cattle and 24.7 million head of sheep, making NSW the largest producer of sheep and the Australian state with the greatest number of cattle and sheep transacted at saleyards (Meat and Livestock Australia 2022c). Saleyards in Australia are publicly or privately owned venues where livestock are assembled for sale and purchase. Types of sales include: ‘fat sales’, selling animals for direct slaughter; ‘store sales’, selling animals not ready for slaughter and requiring ‘finishing’; ‘stud sales’, animals to be used as breeders; and bobby (surplus) calf sales (AUSVETPLAN 2021). About 14.5 million sheep and 4 million cattle pass through saleyards annually, making them the most common livestock species at saleyards (Pitt 2024).

Before livestock is transported to saleyards, the Australian National Livestock Identification System (NLIS) requires that all cattle, and sheep born after 1 January 2025 are individually identified with a radio-frequency identification device (RFID) and registered to the unique Property Identification Code (PIC) of the property where the animal was born. Properties, saleyards, feedlots, processors (abattoirs), pre-export assembly depots, and ports of export are also identified with a unique PIC. When cattle and sheep are moved from one property to another, the move must be recorded in the NLIS database either by close of business for saleyards and abattoirs, or within 48 h by owner or agent for private movement/sales (Moore *et al.* 2015). Using the data of the NLIS from 2008–2012 for the movement of cattle, Iglesias and East (2015) reported that approximately 85% of producers send at least one consignment to a saleyard annually, with approximately 40% of the Australian cattle passing through a saleyard each year. However, these figures should be interpreted with caution, as producer compliance is not 100% (Marriot *et al.* 2025).

Transiting through a saleyard can be stressful and impinge upon animal welfare (Bravo *et al.* 2024). Since livestock experience varying degrees of handling on-farm and during transport prior to arriving at saleyards, these pre-saleyard conditions may influence the animals’ health, welfare, and behavioural or physiological responses to handling upon arrival (Department of Economic Development 2018). Transport exposes animals to multiple challenges — including fatigue, dehydration, injury, and fear — especially when vehicle design, driving conditions, or animal fitness for transport are inadequate (Mench 2004; Collins *et al.* 2018; Nielsen *et al.* 2022a,b). Transporting unfit animals is a major welfare concern, often resulting in deaths on arrival (DOA) (Grandin 2001; Cockram 2019), downers (i.e. livestock unable to walk away from the vehicle), or animals unfit for sale and euthanased at the saleyard (Department of Economic Development 2018). Therefore, animals arriving at the saleyards should be provided with the necessary requirements to meet strict welfare standards, including the provision of appropriate nutrition, availability of sufficient water, enough space (to stand, lie down, and stretch), appropriate handling, and protection from extreme weather (Department of Economic Development 2018). According to an NGO report (Animals Angels 2024), there are significant welfare challenges at Australian saleyards, including overcrowded pens, lack of water and feed, absence of shade, lack of prompt treatment for injuries and disease, inappropriate loading and unloading, inadequate journey conditions before arrival, and sale of unfit animals. These are the risk factors believed by the NGO to increase the mortality of livestock in Australian saleyards (Animals Angels 2024); however, to date, there have yet to have been any scientific studies examining mortality at saleyards and only very few have investigated animal welfare in this or similar contexts (Gregory *et al.* 2009; Bravo *et al.* 2019, 2020, 2024; Wilson *et al.* 2020). Nevertheless, mortality rates have been reported on-farm and during transport, providing valuable insights for comparison. Beggs *et al.* (2019) reported an annual mortality rate of 1.9% for dairy cattle after studying 50 farms in Western Victoria. In breeder beef farms, the annual mortality rate was estimated to range from 3 to around 12% in northern Australian beef operations (Henderson *et al.* 2013). For sheep, the annual mortality rate of adult ewes has been recorded at 4.7%, based on data from 32 farms in Victoria (Doyle 2018), while a survey in NSW reported the annual mortality rate of lambs to be 10% (Kopp *et al.* 2020). A study conducted from 1998 to 2000 in the months of August, September, and October each year in Northern Victoria, Australia, reported a 0.64% overall mortality rate in bobby calves transported by road to abattoirs, with higher mortality linked to longer travel distances, occurring primarily during transport rather than after arrival (Cave *et al.* 2005). Land transport mortality rate in Australia has been calculated to be 0.36% for cattle (Fisher *et al.* 2009). In the 1980s, it was reported that almost 1% of sheep died or were rejected/killed during the part of the market chain from farm to the wharf before export by sea (Norris *et al.* 1989). More recently, it was reported that almost 3% of sheep are affected by a welfare issue upon arrival at an export slaughterhouse (Carnovale *et al.* 2025).

Based on these reports, we hypothesised that transport and marketing practices increase the risk of mortality in comparison with that recorded on farms, and that the sale mortality rate would be associated with environmental, transport and saleyard conditions. Sale mortality was defined as DOA, plus dead or euthanased during time at the saleyard. The current research aimed therefore to determine sale mortality rate from 2021 to 2024 in a sample of saleyards for cattle and sheep in NSW (i.e. the Australian state with the highest sale transactions) and identify possible factors that may increase or decrease this risk via retrospective analysis of NLIS data.

## Materials and methods

### Data extraction and handling

Livestock data between January 2021 and December 2024 from all 69 saleyards located in NSW were downloaded by Authorised NSW Department of Primary Industry and Regional Development (DPIRD) Officers from the NLIS database. At saleyards, the electronic IDs for individually identified animals are scanned on arrival, and saleyard staff must report in the NLIS database those which are deceased, without differentiating between DOA and deceased at the saleyards (DAS). As mandatory individual identification was required for cattle in the examined period but not for sheep, the data regarding all cattle saleyards (n = 44) were retained as downloaded. However, for sheep, data from only four saleyards were utilised, as these were considered by the NSW animal welfare officers of the DPIRD to record deceased animals accurately. The following data were extracted into an Excel® sheet for each sale: saleyard location (coded for privacy); date of sale; region; species (i.e. cattle or sheep); number of deceased animals (i.e. dead-on-arrival, and during their time at the saleyard); and number of animals present at the saleyard. To test for the effect of the number of livestock present in the saleyard on mortality rate, the size of the saleyard was classified (based on the quartile distribution of the number of animals present) in the following four categories: Small (cattle: 3–258; sheep: 1,053–8,602); Medium (cattle: 259–614; sheep: 8,603–12,065); Large (cattle: 615–1,205; sheep: 12,066–24,818); and Very Large (cattle: 1,206–10,771; sheep: 24,819–79,986). The date of sale was separately coded for day, month, year, and season.

To incorporate environmental factors, daily maximum and minimum temperatures of the saleyard location were retrieved from the Australian Government Bureau of Meteorology (BOM) website (Bureau of Meteorology 2025) for each day of sale, before coding the location. Minimum daily temperature was included only for sheep, as it has been reported that cattle are often less prone to cold stress than sheep, especially shorn sheep and lambs (Nielsen *et al.* 2022b; Masters *et al.* 2023; Tüfekci & Sejian 2023).

The cleaning of the data accounted for instances where there were several lines for the same sale date, as some animals arrived before the sale or others were recorded after the sale date. These data were combined, to make sure to have a single entry, reporting the total number of animals sold and deceased for each sale day. The % mortality rate for each species at each sale was calculated using the formula (Thrusfield 2018):

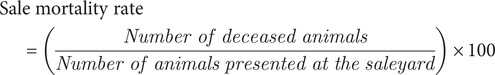



### Statistical analysis

Descriptive statistics were obtained using the SPSS® 25 statistical software package (SPSS® Inc, Chicago, IL, USA), and data were described as means, standard deviation (SD), minimum values, maximum values, median, and interquartile ranges. Univariable and multivariable linear regression models were created to test for the effects of region, saleyard location, year, season, month, size of saleyard, and maximum daily temperature on the sale mortality rate for cattle and sheep. For sheep, the minimum daily temperature was included as a predictor (Table 1). To reduce the impact of extreme values on the analysis, observations below the 2^nd^ percentile and above the 98^th^ percentile of the sale mortality rate distribution were excluded from the regression analysis.

**Table 1.** Name, definition, type and role of variables included in the regression analysis of cattle sale mortality rates

Name	Definition	Type of variable	Statistical role
Maximum daily temperature	The maximum daily temperature at the saleyard location	Numerical	Predictor
Minimum daily temperature	The minimum daily temperature at the saleyard location	Numerical	Predictor (only for sheep)
Year	The year of sale	Categorical	Predictor
Month	The month of sale	Categorical	Predictor
Season	The season when the saleyard was performed, calculated using equinoxes and solstices	Categorical	Predictor
Size of saleyard	The size of the saleyard based on the number of animals present at the saleyard	Categorical	Predictor
Region	The region where the saleyard was performed	Categorical	Predictor
Saleyard location	The coded location where the saleyard was performed	Categorical	Predictor
Species	The species of animals present at the saleyard (cattle or sheep)	Categorical	Predictor (only for sale mortality rate)
Sheep sale mortality rate	Total number of dead sheep on a given day/the total number of sheep present that day × 100	Numerical	Outcome
Cattle sale mortality rate	Total number of dead cattle on a given day/the total number of cattle present that day × 100	Numerical	Outcome

For the univariable analysis, separate linear regression models were created to assess the association between sale mortality rate and each predictor, for cattle and sheep separately. Their significance was determined using *t*-tests to compare each category against the reference and the F-test to assess the overall fit of the model (Fox 2015). In the analysis of each outcome, the variables with a *P*-value < 0.100 were initially retained for inclusion in the backwards stepwise selection for multivariable linear regression models. In both the multivariable regression analyses, variance inflation factors (VIFs) were used to evaluate multicollinearity among the independent variables included in the models. Variables with a VIF score higher than 5 were considered to be collinear (Menard 2001). The variables ‘region’ and ‘saleyard location’ were collinear. Therefore, only saleyard location was retained for inclusion in the multivariable linear regression models, based on a model selection process using the Akaike Information Criterion (AIC). Backwards elimination was conducted manually, with variables sequentially removed until only those with a *P*-value ≤ 0.10 remained in the final model for each species. Observations with missing values were automatically excluded from the analysis by the software, and the categories used as references were chosen as they were those with the lowest mortality, to facilitate the interpretation of the estimates. Furthermore, the cattle and sheep data-sets were merged to assess the relationship between species (cattle vs sheep) and sale mortality rate, with species included as a predictor in a linear regression model. Regression analysis was conducted in R environment (R Version 4.4.3; www.r–project.org) and significance was set at *P* < 0.05. Trends toward significance were set at 0.10 > *P >* 0.05.

## Results

The summary descriptive statistics of the numerical and categorical variables are reported in Tables S1, S2 and S3 (see Supplementary material). Between 2021–2024, an average of 937.4 cattle were present on each day of sale across NSW saleyards, while the average number of sheep present each day of sale, calculated from the four locations for which data were retained, was 19,646.8. On average, almost 20 sheep died at each sale, while less than one head of cattle died. The maximum numbers of sheep and cattle dead on any sale were 641 (out of 55,976 animals present at the sale) and 18 (out of 2,415 animals present at the sale), respectively. The mean and maximum temperatures were similar for the two species; mean temperatures were 23 and 22°C for cattle and sheep, respectively, while maximum temperatures were 43 and 41°C, respectively. Minimum temperature was –4.7°C for sheep (Table S1; Supplementary material). The distribution of saleyard days was consistent across years, months, seasons, and size, but sale days were unevenly distributed across saleyards (Tables S2 and S3; Supplementary material). The number of cattle and sheep present at each sale day varied with study period and is presented, stratified by region, saleyard location, month, year, season, and size of the saleyards in Tables S4 and S5 (Supplementary material).

### Cattle mortality across saleyards

**Figure 1.**
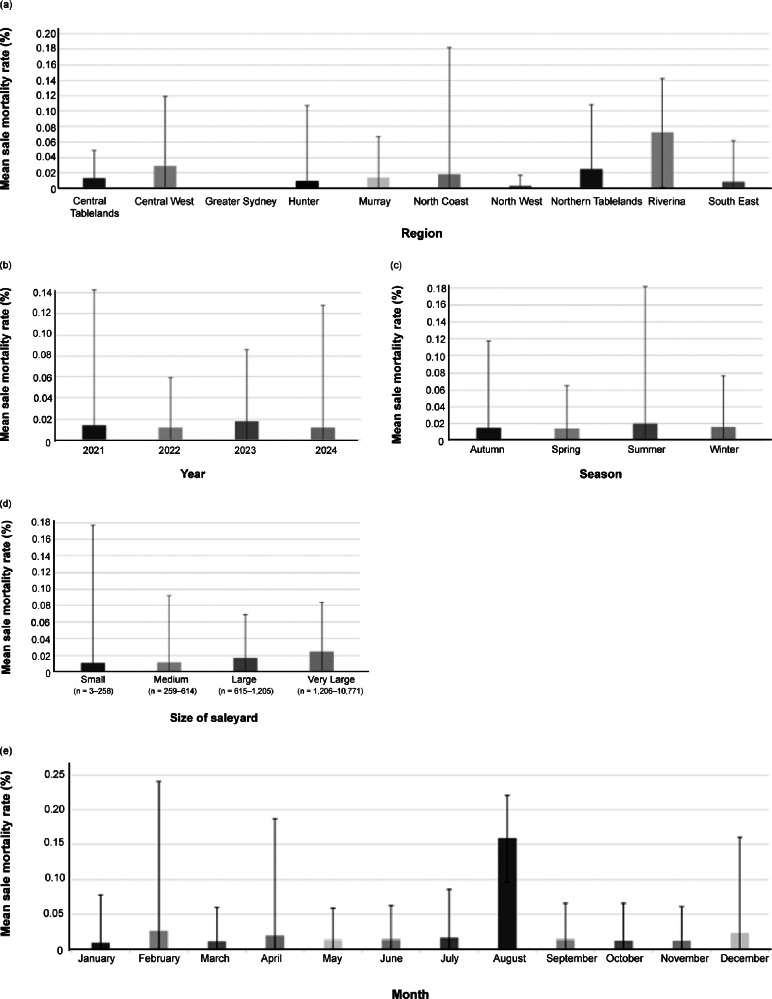
Mean sale mortality rate (%) for cattle registered in the National Livestock Identification System from 2021 to 2024 at all saleyards in NSW (Australia) stratified by (a) region, (b) year, (c) season, (d) size of saleyard, and (e) month. Error bars indicate standard deviation (SD).

The overall mean cattle sale mortality rate in 2021–2024 was 0.016%. Figure 1 shows the mean cattle sale mortality rate stratified by region, year, month, season, and size of saleyard. There was considerable regional variation: the Riverina region recorded the highest mean mortality rate (0.072%), while the Northwest region recorded the lowest (0.003%). The maximum sale mortality rate was recorded in the North Coast, at 4.76%, and the minimum sale mortality rate was zero for all regions (Figure 1). The overall sale mortality rate and the descriptive statistics for the different saleyard locations are shown in Table S6 (Supplementary material).

The univariable regression models show that the cattle sale mortality rate was positively associated with maximum daily temperature (*P* = 0.009), year (*P* = 0.001), and size of saleyard (*P* < 0.001). It was higher in 2023 (*P* = 0.003) and 2024 (*P* = 0.001) than in 2021, and Large and Very Large saleyards had a higher mortality rate in comparison with Small saleyards (*P* < 0.001). Additionally, there was a trend towards significantly different mortality rates for different months (*P* = 0.072). There was no significant effect of region, but there was an effect of the individual saleyard location (*P* < 0.001) (Table 2; Table S7 [Supplementary material]).

**Table 2.** Univariable linear regression models assessing the association between cattle sale mortality rate and the variables: maximum daily temperature, year, month, season, and size of saleyard

Variable	Estimate (± SE)	(95% CI)	*P*–value
**Maximum daily temperature**	0.0001 (± 0.000)	(0.000, 0.000)	**0.009**
**Region**			**0.106**
Greater Sydney	Ref.		
Central Tablelands	0.011 (± 0.002)	(0.007, 0.016)	< 0.001
Central West	0.016 (± 0.002)	(0.012, 0.020)	< 0.001
Hunter	0.001 (± 0.002)	(–0.003, 0.005)	0.617
Murray	0.005 (± 0.003)	(–0.002, 0.012)	0.133
North Coast	0.007 (± 0.002)	(0.003, 0.011)	< 0.001
North West	0.003 (± 0.002)	(–0.001, 0.007)	0.182
Northern Tablelands	0.012 (± 0.002)	(0.007, 0.016)	< 0.001
Riverina	0.061 (± 0.003)	(0.056, 0.066)	< 0.001
South East	0.003 (± 0.002)	(–0.001, 0.007)	0.106
**Year**			**0.001**
2021	Ref.		
2022	0.001 (± 0.001)	(–0.001, 0.003)	0.451
2023	0.003 (± 0.001)	(0.001, 0.005)	0.003
2024	0.003 (± 0.001)	(0.001, 0.005)	0.001
**Month**			**0.072**
January	Ref.		
February	0.004 (± 0.002)	(0.000, 0.008)	0.027
March	0.003 (± 0.002)	(–0.001, 0.006)	0.140
April	0.001 (± 0.002)	(–0.002, 0.005)	0.491
May	0.005 (± 0.002)	(0.001, 0.008)	0.009
June	0.005 (± 0.002)	(0.001, 0.008)	0.010
July	0.001 (± 0.002)	(–0.002, 0.005)	0.458
August	0.004 (± 0.002)	(0.000, 0.008)	0.031
September	0.004 (± 0.002)	(0.000, 0.008)	0.032
October	0.003 (± 0.002)	(0.000, 0.007)	0.076
November	0.004 (± 0.002)	(0.001, 0.008)	0.024
December	0.006 (± 0.002)	(0.002, 0.010)	0.004
**Season**			0.828
Autumn	Ref.		
Spring	0.001 (± 0.001)	(–0.001, 0.003)	0.353
Summer	0.000 (± 0.001)	(–0.002, 0.002)	0.757
Winter	0.001 (± 0.001)	(–0.001, 0.002)	0.610
**Size of saleyard**			**< 0.001**
Small	Ref.		
Medium	0.002 (± 0.001)	(0.000, 0.004)	0.117
Large	0.011 (± 0.001)	(0.009, 0.013)	<0.001
Very Large	0.020 (± 0.001)	(0.018, 0.022)	< 0.001

*P*-values in bold refer to the statistical significance of the predictive variable in the model; the significance of a category in comparison with the reference value is reported in regular font.

CI = confidence interval; Ref. = reference category; SE = standard error.

The variables, year (*P* < 0.001), month (*P* = 0.023), size of saleyard (*P* < 0.001) and saleyard location (*P* < 0.001) were significant in the multivariable linear regression model for cattle sale mortality rate (model: *P* < 0.001, AIC: –26881.69). The months of May (*P* = 0.029), June (*P* = 0.020), September (*P* = 0.026), and December (*P* = 0.011) were associated with higher cattle mortality rates compared to January, the reference month (Table 3).

**Table 3.** Multivariable linear regression model results for the outcome variable cattle sale mortality rate

Variable	Estimate (± SE)	(95% CI)	*P*–value
**Year**			**< 0.001**
2021	Ref.		
2022	0.000 (± 0.001)	(–0.002, 0.002)	0.992
2023	0.003 (± 0.001)	(0.001, 0.005)	0.002
2024	0.003 (± 0.001)	(0.001, 0.005)	0.003
**Month**			**0.023**
January	Ref.		
February	0.002 (± 0.002)	(–0.001, 0.003)	0.135
March	0.001 (± 0.002)	(–0.002, 0.002)	0.529
April	0.000 (± 0.002)	(–0.003, 0.003)	0.960
May	0.004 (± 0.002)	(0.000, 0.007)	0.029
June	0.004 (± 0.002)	(0.001, 0.007)	0.020
July	0.000 (± 0.002)	(–0.003, 0.004)	0.850
August	0.003 (± 0.002)	(–0.001, 0.007)	0.051
September	0.004 (± 0.002)	(0.000, 0.007)	0.026
October	0.003 (± 0.002)	(0.000, 0.006)	0.073
November	0.003 (± 0.002)	(0.000, 0.007)	0.053
December	0.005 (± 0.002)	(0.001, 0.009)	0.011
**Size of saleyard**			**< 0.001**
Small	Ref.		
Medium	0.001(± 0.001)	(–0.001, 0.003)	0.482
Large	0.007 (± 0.001)	(0.004, 0.009)	< 0.001
Very Large	0.006 (± 0.001)	(0.004, 0.009)	< 0.001
Saleyard location^*^			**< 0.001**
1	Ref.		
2	0.001 (± 0.005)	(–0.009, 0.010)	0.857
3	0.006 (± 0.003)	(0.000, 0.012)	0.031
4	–0.001 (± 0.008)	(–0.016, 0.015)	0.934
5	–0.002 (± 0.004)	(–0.010, 0.006)	0.633
6	0.001 (± 0.002)	(–0.004, 0.006)	0.704
7	0.012 (± 0.002)	(0.007, 0.016)	< 0.001
8	0.023 (± 0.002)	(0.018, 0.027)	< 0.001
9	–0.001 (± 0.004)	(–0.009, 0.007)	0.864
10	0.001 (± 0.004)	(–0.007, 0.009)	0.793
11	0.001 (± 0.004)	(–0.007, 0.009)	0.791
12	0.000 (± 0.003)	(–0.006, 0.007)	0.924
13	0.018 (± 0.002)	(0.013, 0.022)	< 0.001
14	–0.002 (± 0.003)	(–0.007, 0.004)	0.539
15	0.000 (± 0.005)	(–0.009, 0.007)	0.927
16	0.004 (± 0.003)	(–0.002, 0.011)	0.162
14	0.020 (± 0.002)	(0.015, 0.024)	< 0.001
17	0.005 (± 0.008)	(–0.011, 0.022)	0.529
19	–0.002 (± 0.003)	(–0.008, 0.004)	0.469
20	0.001 (± 0.002)	(–0.003, 0.005)	0.613
21	–0.006 (± 0.011)	(–0.028, 0.016)	0.608
22	–0.001 (± 0.002)	(–0.006, 0.003)	0.718
23	0.014 (± 0.002)	(0.010, 0.019)	< 0.001
24	–0.002 (± 0.002)	(–0.007, 0.003)	0.389
25	–0.002 (± 0.003)	(–0.007, 0.003)	0.459
26	0.000 (± 0.003)	(–0.006, 0.005)	0.901
27	0.002 (± 0.002)	(–0.003, 0.006)	0.450
28	0.002 (± 0.007)	(–0.012, 0.015)	0.807
29	0.000 (± 0.002)	(–0.005, 0.005)	0.968
30	–0.003 (± 0.003)	(–0.008, 0.003)	0.329
31	0.000 (± 0.003)	(–0.006, 0.007)	0.911
32	–0.001 (± 0.004)	(–0.009, 0.007)	0.864
33	0.002 (± 0.006)	(–0.009, 0.013)	0.751
34	0.001 (± 0.003)	(–0.003, 0.007)	0.608
35	–0.003 (± 0.002)	(–0.007, 0.001)	0.241
36	–0.001 (± 0.002)	(–0.005, 0.003)	0.749
37	–0.001 (± 0.002)	(–0.006, 0.003)	0.557
38	0.001 (± 0.003)	(–0.004, 0.007)	0.727
39	0.000 (± 0.003)	(–0.006, 0.006)	0.955
40	–0.004 (± 0.011)	(–0.026, 0.019)	0.744
41	0.059 (± 0.003)	(0.054, 0.065)	< 0.001
42	0.001 (± 0.003)	(–0.005, 0.006)	0.785
43	0.000 (± 0.010)	(–0.020, 0.021)	0.967
44	0.007 (± 0.002)	(0.003, 0.011)	0.002

*P*-values in bold refer to the statistical significance of the predictive variable in the model; the significance of a category in comparison with the reference value is reported in regular font.

CI = confidence interval; Ref. = reference category; SE = standard error.

* Saleyard locations have been coded for privacy.

### Sheep mortality rate across saleyards

**Figure 2.**
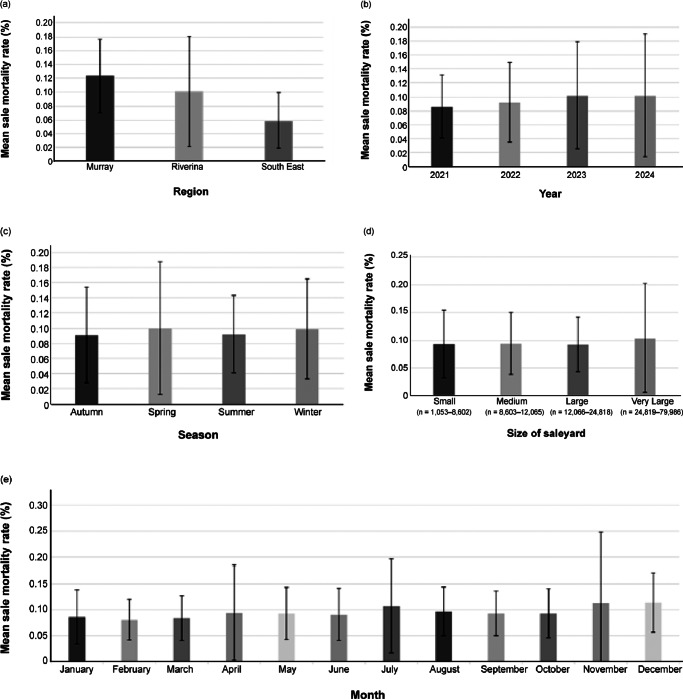
Mean sale mortality rate (%) of sheep registered in the National Livestock Identification System from 2021 to 2024 at four saleyards in NSW (Australia) stratified by (a) region, (b) year, (c) season, (d) size of saleyard, and (e) month. Error bars indicate standard deviation (SD).

The overall sale mean mortality rate for sheep was 0.0957%, with values stratified by region, year, month, season, and size of saleyard shown in Figure 2. The maximum mortality rate was registered in the Riverina region in November 2024 (1.145%). The descriptive statistics stratified by location of saleyard are presented in Table S8 (see Supplementary material).

In univariable regression models, the sale mortality rate was associated with region (*P* < 0.001) and saleyard location (*P* < 0.001). The saleyards located in the Murray and Riverina regions had higher mortality rates compared to the South-East (*P* < 0.001). Similarly, saleyards 2, 3, and 4 had higher mortality rates than saleyard 1 (*P* < 0.001). A significant negative relationship was found with minimum daily temperature (*P* = 0.004), indicating that lower minimum temperatures were associated with higher sheep mortality rates. No other significant associations were found. The variables retained in the final multivariable linear regression model for sheep sale mortality rate were minimum daily temperature and saleyard location (model: *P*-value < 0.001, AIC: –2,569.41) (Table S9; Supplementary material).

### Effect of species on sale mortality rate

The sale mortality rate for sheep (0.0957%) was higher than that for cattle (0.0160%) (*P* < 0.001) (Sheep vs Cattle, estimate = 0.079, SE = 0.001, 95% CI = 0.076–0.081).

## Discussion

Our study documented the mean mortality rates of cattle (0.016%) and sheep (0.096%) recorded during sale days in a sample of Australian saleyards, based on data extracted from the NLIS database, and highlighted the possible factors associated with these outcomes, as well as potential limitations related to the data entry methods of the database itself.

We demonstrated higher mortality of sheep compared with cattle at saleyards, although caution is required regarding interpretation, since the results for sheep originated from four selected saleyards, while for cattle results were from all 44 saleyards in the state. Nevertheless, the magnitude of the difference, with sheep mortality being eight times higher than that of cattle, suggests a credible distinction between the two. The higher mortality observed in sheep compared to cattle may be related to several factors. Sheep’s apparent resilience, reflecting their prey-animal tendency to mask signs of pain or fatigue, may conceal underlying distress and delay intervention (Steagall *et al.* 2021). Their larger numbers at saleyards may also increase risks associated with overcrowding, exposure to unfamiliar animals, and reduced individual attention from handlers, although this was not directly assessed. Moreover, sheep in Australia are generally managed under extensive systems with limited human contact (Muñoz *et al.* 2019), making the sudden confinement and frequent handling particularly stressful. Such conditions may compromise immune function (Sutherland *et al.* 2019) and contribute to greater morbidity and mortality in sheep. Moreover, this might be due to the lower economic value of sheep compared to cattle, resulting in comparatively less provision for their welfare. Further studies are needed to confirm those hypotheses, collecting data along the chain from the farm to the saleyard.

Higher mortality rate in sheep in comparison with cattle is consistent with reports from Australian export slaughterhouses (Carnovale *et al.* 2025) and an Italian control post, where mortality was also associated with overcrowding (Padalino *et al.* 2018). However, it is worth highlighting that in our study, sheep mortality was three times higher than reported for sheep transported and stopped at intermediate facilities in Europe (Padalino *et al.* 2018). This might be due to differences in husbandry practices (e.g. mulesing; Lee & Fisher 2007), vehicle types — including open-top trucks without controlled ventilation, which are allowed in Australia (Fisher *et al.* 2009) — and different management before, during and after the journey, all of which may further compromise the health status of sheep arriving at Australian saleyards. However, as these factors have not been directly addressed by our study, further research is needed to investigate them.

The sale mortality rate was calculated as the ratio between the deceased animals and the number of animals present at the sale date. However, the deceased animals were the sum of DOA, animals killed as judged unfit for sale or to continue to travel, and animals who died at the saleyards. The cause of death is consequently multifactorial, and the effects of the journey duration and conditions cannot be separated. During land transportation of livestock, factors such as loading and handling practices, the transport environment, feed and water deprivation, truck physical and thermal conditions, and journey duration may all affect animal welfare (Nielsen *et al.* 2022a,b). Fitness for transportation is another important aspect that should be considered to avoid unwanted consequences (Cockram 2019; Nielsen *et al.* 2022b). In Australia, animals that are emaciated or weak, have clinical conditions, respiratory disorders, impaired locomotion, or are unable to see/blind are considered unfit for transportation (Department of Economic Development 2018). It is therefore reasonable to conclude that the factors contributing to the death of cattle and sheep registered at the saleyards may be the combination of many associated factors along the entire chain; however, targeted studies would be necessary to better understand and confirm their influence.

Scaled up (multiplying sale day mortality by 365 days, to calculate annual equivalent mortality), sheep and cattle showed annual equivalent mortality rates of 34.9 and 5.8%, respectively. For sheep, this is approximately five times greater than that reported for on-farm, yearly mortality in Victoria (4.7%) (Doyle 2018) and 3.5 times greater than that recorded for lambs in NSW (10%) (Kopp *et al.* 2020). For cattle, this is within the range of beef cattle mortalities reported on-farm (3–12%) (Henderson *et al.* 2013), but greater than the mortality reported for dairy cattle (1.9%) (Beggs *et al.* 2019). This emphasises the greater risk to sheep mortality compared to cattle when they are travelling to and being kept in saleyards. Sheep with high level of wool cover of their body could be challenged by transportation in a hot climate, especially when the truck is stopped for longer periods during the journey (Fisher *et al.* 2009). This may lead to panting and dehydration, causing sheep to suffer more than cattle (Fisher *et al.* 2009). However, according to an advocacy group (Animals Angels 2011), the frequent transportation of diseased or unfit sheep seems to be another contributing factor to the increased sheep sale mortality. Chronically diseased and weak sheep frequently arrive in poor health, necessitating emergency slaughter or dying soon after arrival (Carnovale *et al.* 2025). This scenario might help to explain higher sheep mortality in this context; however, our study could not specifically investigate these aspects due to a lack of information. Further studies are needed to assess the welfare of sheep at the saleyards and along the entire supply chain, in order to better understand potential welfare hazards.

The increased mortality of cattle in Large and Very Large saleyards may be caused by several factors. Less attention to individual animal welfare at larger saleyards is possible, but animals might also experience longer delays in loading and unloading, which are well-known hazards to cattle welfare during transport (Maria *et al.* 2004). At bigger saleyards, animals might also be more stressed by the presence of many unfamiliar conspecifics (Šímová *et al.* 2016). Mixing with unfamiliar animals increased the negative welfare indicators in a study conducted in Chile, where the behaviour of calves in 12 auction markets was analysed (Bravo *et al.* 2019). In Large and Very Large saleyards, animals may face other stressors, such as overcrowding, fighting for resources, excessive physical restraint, injuries, loud noise, inappropriate driving (Bravo *et al.* 2019) and exposure to various pathogens (Nielsen *et al.* 2022a,b). Consequently, adapting to a large, novel and challenging environment may be difficult, especially after a long journey (Valadez-Noriega *et al.* 2022). In particular, post-transport fatigue and exposure to the pathogens causing the development of respiratory disease related to transport (i.e. shipping fever), as well as the stress that has been observed in feedlot studies, could contribute to increasing cattle vulnerability upon arrival at the Large and Very Large saleyards (Valadez-Noriega *et al.* 2022). It may also be possible that managers of Large and Very Large saleyards report more systematically and reliably. A prospective study is needed to confirm this finding.

Based on our analysis, our data-set indicates that the number of cattle present in a saleyard varied across years and months. The fact that the number of animals sold at saleyards was higher in some years and months might be one reason for the association between cattle mortality rate and specific time-periods. Notably, cattle sale mortality was higher in 2023 and 2024, when the number of animals sold was also greater (more than 1,000 animals in 2023 and 2024, around 800 in 2022, and just 750 in 2021; Table S4 [see Supplementary material]. In 2021 and 2022, fewer cattle were present at saleyards, possibly due to the COVID-19 pandemic, which significantly impacted the beef supply chain (Meat and Livestock Australia 2022b). Another possible factor is the occurrence of drought in 2018–2019, which led to a reduced beef population for several years (Windsor 2021). Similarly, the higher cattle sale mortality rate in May, June, September, and December compared to January might be due to the increased number of animals passing through the saleyards during these months (Table S4; Supplementary material). In Australia’s southern beef production system, the period from July to September is typically when young stock are sent to market. Conversely, in January, the lowest number of animals is sold, as it is the summer holiday period (Meat and Livestock Australia 2022a).

Monthly differences in sheep sale mortality were not significant, except between January and December which, similar to cattle, could be due to the reduced activity and fewer working days during the holiday season. Peaks in the number of sheep passing through saleyards were observed during spring (September–November), when the greatest number of young lambs entered the market, and around religious holidays, such as Orthodox Easter and Eid al-Fitr, which increased supply and saleyard activity due to higher export demands (Department of Agriculture Fisheries and Forestry 2025; Meat and Livestock Australia 2025). Lambs, in particular when travelling after weaning, with few stopovers during transportation, little access to food and with limited space allowance, are more prone to transport and new environment stress, resulting in a higher incidence of health issues due to a compromised immune system (Cockram *et al.* 1996; Tadich *et al.* 2009; Miranda‐de la Lama *et al.* 2012; Nielsen *et al.* 2022b). Our data are in line with other studies suggesting that when there is movement of young animals under time pressure, the risk of mortality increases (Padalino *et al.* 2018; Angeloni *et al.* 2025). Therefore, during peak periods, greater attention should be given to ensuring that handling, transportation, and management measures for large numbers of animals at saleyards are properly implemented and observed.

The significant increases in cattle mortality at high temperatures and sheep mortality at low temperatures in the univariable models were small in magnitude but emphasise the risk of temperatures outside the species-specific thermoneutral zone. However, our results should be interpreted with caution because the temperature data were downloaded from a website for the location, so they do not represent the real temperature the animals were exposed to during the journeys and at the saleyards. Notwithstanding those limitations, our preliminary findings confirm that both extreme cold and hot temperatures may cause thermal stress resulting in the impairment of the physiological status of animals, potentially leading to mortality (Masters *et al.* 2023). Sheep, being smaller in size compared to cattle are often more prone to cold stress, especially if young or recently shorn (Maurya *et al.* 2013; Tüfekci & Sejian 2023). Conversely, cattle are more likely to find high temperatures stressful and will quickly succumb to temperature stress if unable to lose body heat (Mader *et al.* 2006; Burhans *et al.* 2022). Provision of adequate, clean, fresh water is essential (Kamphues 2000). However, according to reports from an advocacy group, water is not always available in saleyard pens (Animals Angels 2024), and even when it is, actual consumption may be limited by inappropriate drinker design (Machado Filho *et al.* 2004) or high water temperature (Savage *et al.* 2008; Menchetti *et al.* 2021). Shading drinking points at animal markets, depots and saleyards is therefore recommended (Department of Economic Development 2018; Zappaterra *et al.* 2021). Also, access to shelter and shade is crucial for the animals, as it helps to mitigate the effects of extreme temperatures and wind, reduce heat stress, and improve welfare (Masters *et al.* 2023). Some saleyards provide shelter or shade, while others do not, leading to variability in welfare conditions across individual saleyards (Animals Angels 2024; DPIRD vet personal communication, 2025). Unfortunately, in our analysis, the presence/absence of shelters in each saleyard was not made available, and this may have affected our findings. Further studies considering the actual environmental conditions that animals are subjected to during the sale day and the access to shade should be conducted.

The effects of the individual saleyard could be interpreted in several ways. It could be related to the facilities (presence of shelter), the different management (feeding and watering), the data recording method (more or less accurate), the handling methods (more rough/gentle) (Bravo *et al.* 2020). This was found in a previous study, where the different designs in races and gates, and handling equipment resulted in different carcase bruising in cattle (Weeks *et al.* 2002). However, it may also be due to the type of animals (McNally & Warriss 1997) and the journey conditions and durations (Durr *et al.* 2009) they are subjected to prior to arriving at a specific saleyard. The level of conscientiousness in data recording is not known. It is worth highlighting that several cattle saleyards recorded zero deceased animals over four years, which requires verification. This suggests a need to train people in entering data into the NLIS systems. Data entries need to be double-checked regularly, and anomalous data flagged. Mortality is a good iceberg indicator of welfare; therefore, knowing that from 1 January 2025, all sheep and farmed goats will be required electronic identification for movement away from the property of birth, we suggest that the NLIS sale mortality data should be regularly checked. This practice could help the DPI and the animal welfare officers to open audits in the saleyard locations that need more attention. In many countries, such as those in the European Union and in Canada, there are suggested thresholds of mortality for DOA which necessitate monitoring by competent authorities, and higher DOA are investigated (Council of the European Union 2005; Government of Canada 2020).

### Study limitations

Our study emphasises the mortality risk for sheep at saleyards, but it is important to be cautious due to the small sample size of the sheep saleyards and the limited mortality returns. This was one of the major limitations of the study. The other major limitation is that this study is based on actual data analysis, with all the inherent limitations of data entry in the NLIS. In particular, regarding NLIS systems, there is no possibility of distinguishing between DOA, animals’ euthanased and animals found dead at the saleyards. This made it impossible to distinguish between the possible effects of inappropriate journey conditions and saleyard-related factors. Another limitation is that the causes of death and the animal classes (i.e. age, sex, breed, physiological status) were not reported in the NLIS and could not subsequently undergo analysis. We recommend that data recording should include a division of deceased animals (i.e. DOA, euthanased at the saleyard, or died at the saleyard), and possibly the animal class, or the type of sales, to enable more detailed future studies and, above all, to ensure better monitoring and protection of animal welfare. Additionally, the data entry should be implemented as soon as possible with sheep. We also identified several data inaccuracies, highlighting the requirement for better training of saleyard staff responsible for data entry to ensure accurate and consistent records. Finally, regarding the environmental conditions, apart from the aforementioned, it is worth noting that the minimum temperature was analysed only for the sheep. Notwithstanding these limitations, this is the first study to provide evidence on mortality along the entire saleyard-related chain — from loading at the farm, during transportation, to re-loading at the saleyards — in NSW, Australia, confirming that passing through auction markets may compromise livestock welfare (Bravo *et al.* 2024).

### Animal welfare implications

Animals travelling to and staying at saleyards are at a higher risk of death compared with when they are kept on farms (Doyle 2018; Kopp *et al.* 2020). Therefore, we confirmed that travelling to and staying at saleyards should be considered welfare hazards (McNally & Warriss 1997; Weeks *et al.* 2002; Bravo *et al.* 2024). To minimise the risk of mortality, livestock should be fit for transport (Mench 2004; Collins *et al.* 2018), travel in adequate conditions (WOAH 2024) and be provided with the minimal welfare standards at all saleyards, such as appropriate feed, adequate water, protection from extreme weather, low stress handling, and adequate space allowance (Department of Economic Development 2018). As previously suggested (Bravo *et al.* 2020), all stakeholders involved in the cattle and sheep production chain should undergo training on minimal welfare standards, and in Australia, ad hoc training should be offered to the people involved in data entry in the NLIS system.

## Conclusion

This study documented, for the first time, the mortality rate registered at cattle and sheep saleyards in a sample of saleyards in Australia. The mortality rate of sheep was eight times that of cattle, suggesting that the welfare of sheep through the marketing chain might be at higher risk. However, the study also showed a significant individual effect of saleyard. The reasons for this effect cannot be fully determined from this study, but possible contributing factors might include the type of animal and their journey conditions while they reach a specific saleyard, the management of the animals at that saleyard, but also the data recording method. Several inaccuracies were found in the data; therefore, we recommend that saleyard staff responsible for data entry should be better trained in this process. Further and larger prospective studies are needed to investigate the causes of death in sheep and cattle at saleyards and their risk factors and confirm our preliminary findings. Such studies will be possible addressing a number of the limitations highlighted in the current study .

## Supporting information

10.1017/awf.2025.10058.sm001 Padalino et al. supplementary material Padalino et al. supplementary material

## Supplementary material

The supplementary material for this article can be found at http://doi.org/10.1017/awf.2025.10058.
