# Supplementary material for: Data analysis of the mortality of cattle and sheep recorded in a sample of Australian saleyards
Source: Anim Welf. 2026 Jan 15;35:e2. doi: 10.1017/awf.2025.10058 (PMC12817224; doi:10.1017/awf.2025.10058)
Supplement: Padalino et al. supplementary material [file S0962728625100584sup001.pdf]

Data analysis of the mortality of cattle and sheep recorded in a sample of Australian saleyards: Supplementary material

Barbara Padalino https://orcid.org/0000-0002-7630-8285[1,2], Naod Thomas Masebo[1], Maria Gaia Angeloni[1] and Clive Julian Christie Phillips[3, 4]

[1] Department of Agricultural and Food Sciences, University of Bologna, 40127 Bologna, Italy

[2] Faculty of Science and Engineering, Southern Cross University, Lismore, NSW, Australia

[3] Curtin University Sustainability Policy Institute, Kent St, Bentley, WA 6102, Australia

[4] Institute of Veterinary Medicine and Animal Sciences, Estonian University of Life Sciences, Kreutzwaldi 1, 51006 Tartu, Estonia

Author for correspondence: Barbara Padalino, email: barbara.padalino@scu.edu.au/barbara.padalino@unibo.it

Table S1. Descriptive statistics of the numerical variables related to cattle and sheep sale days registered in the National Livestock Identification Service (2021–2024) at all cattle saleyards and four sheep saleyards located in New South Wales, Australia.

Variable Name	Mean (± SD)	Minimum	First Quartile	Median	Third Quartile	Maximum
**Cattle (n = 6,046)***						
Maximum daily temperature (°C) [a]	22.1 (± 6.5)	3.0	17.5	22.0	26.9	43.0
Number of Cattle present at saleyard (n)	937.4 (± 1,024.7)	3.0	258.0	614.0	1,205.0	10,771.0
Deceased Cattle (n)	0.23 (± 0.95)	0	0	0	0	18.0
Cattle sale mortality rate (%)	0.016 (± 0.10)	0	0	0	0	4.8
**Sheep (n = 784)***						
Maximum daily temperature (°C) [a]	22.5 (± 7.1)	7.9	16.6	21.8	28.1	41.1
Minimum daily temperature (°C) [a]	8.9 (± 6.17)	-4.7	4.2	8.5	13.8	25.3
Number of Sheep present at saleyard (n)	19,646.8 (± 17,003.8)	1,053.0	8,602.0	12,065.0	24,818.0	79,986.0
Deceased Sheep (n)	19.6(± 31.4)	0	6.0	12.0	24.0	641.0
Sheep sale mortality Rate (%)	0.096 (± 0.1)	0	0.1	0.1	0.1	1.2

SD = Standard Deviation

*The total number of sale days with available data for each species is indicated next to the species name

[a] Missing data: Data were missing for some variables, and the denominator for percentage calculations is based on the number of sale days for which data were available. Data were missing for: Cattle – Maximum daily temperature (°C) (missing = 217, 3.6%); Sheep – Maximum daily temperature (°C) (missing = 15, 1.9%), Minimum daily temperature (°C) (missing = 35, 4.5%)

Table S2. Descriptive statistics of the categorical variables (region, saleyard location, month, year, season, and size of saleyard) from the dataset of 44 cattle saleyards located in NSW (Australia) downloaded from the National Livestock Identification System (NLIS) from 2021 to 2024.

Variable[a]	Category	Count	Percentage (%) of total
**Region**	Central Tablelands	386	6.38
	Central West	711	11.76
	Greater Sydney	200	3.31
	Hunter	1,043	17.25
	Murray	92	1.52
	Northern Tablelands	504	8.34
	North Coast	1,314	21.73
	North West	605	10.01
	Riverina	199	3.29
	South East	992	16.41
**Saleyard location***	1	259	4.28
	2	30	0.50
	3	119	1.97
	4	10	0.17
	5	49	0.81
	6	200	3.31
	7	264	4.37
	8	330	5.46
	9	45	0.74
	10	44	0.73
	11	47	0.78
	12	67	1.11
	13	268	4.43
	14	139	2.30
	15	32	0.53
	16	92	1.52
	17	220	3.64
	18	9	0.15
	19	120	1.98
	20	218	3.61
	21	5	0.08
	22	206	3.41
	23	260	4.30
	24	208	3.44
	25	166	2.75
	26	131	2.17
	27	243	4.02
	28	13	0.22
	29	215	3.56
	30	122	2.02

	31	84	1.39
	32	44	0.73
	33	21	0.35
	34	141	2.33
	35	246	4.07
	36	232	3.84
	37	333	5.51
	38	105	1.74
	39	116	1.92
	40	5	0.08
	41	189	3.13
	42	110	1.82
	43	6	0.10
	44	283	4.68
**Month**	January	401	6.63
	February	544	9.00
	March	576	9.53
	April	537	8.88
	May	591	9.78
	June	540	8.93
	July	493	8.15
	Aguste	529	8.75
	September	497	8.22
	October	501	8.29
	November	523	8.65
	December	314	5.19
**Year**	2021	1,568	25.93
	2022	1,484	24.55
	2023	1,484	24.55
	2024	1,510	24.98
**Season**	Autumn	1,704	28.18
	Spring	1,521	25.16
	Summer	1,259	20.82
	Winter	1,562	25.84
**Size of saleyard**	Small	1,512	25.01
	Medium	1,513	25.02
	Large	1,510	24.98
	Very Large	1,511	24.99

[a]Data are available for a total of 6,046 sale days; no variables had missing values

*Saleyard locations have been coded for privacy

Table S3. Descriptive statistics of the categorical variables (region, saleyard location, month, year, season, and size of saleyard) from the data set of 4 sheep saleyards located in NSW (Australia) downloaded from the National Livestock Identification System (NLIS) from 2021 to 2024.

Variable[a]	Category		Count	Percentage (%) of total
**Region**	Murray		192	24.49
	Riverina		383	48.85
	South East		209	26.66
**Saleyard location***	1		192	24.49
	2		189	24.11
	3		194	24.74
	4		209	26.66
**Month**	January		65	8.29
	February		66	8.42
	March		70	8.93
	April		54	6.89
	May		70	8.93
	June		64	8.16
	July		69	8.80
	Aguste		71	9.06
	September		69	8.80
	October		69	8.80
	November		74	9.44
	December		43	5.48
**Year**	2021		198	25.26
	2022		191	24.36
	2023		197	25.13
	2024		198	25.26
**Season**	Autumn		194	24.74
	Spring		212	27.04
	Summer		174	22.19
	Winter		204	26.02
**Size of saleyard**	Small		196	25.00
	Medium		196	25.00
	Large		196	25.00
	Very Large		196	25.00

[a]Data are available for a total of 784 sale days; no variables had missing values

*Saleyard locations have been coded for privacy

Table S4. Descriptive statistics of the number of cattle heads per sale registered in the National Livestock Identification System (NLIS) from 2021 to 2024 at all saleyards located in NSW (Australia) stratified by region, year, month, season, size of saleyard and saleyard location.

Variable	Category	Number of cattle at each sale		
**Region**		**Mean (± SD)**	**Minimum**	**Maximum**
	Central Tablelands	1,537.55 (± 1,331.87)	27.00	10,771.00
	Central West	1,382.98 (± 1,318.29)	3.00	7,138.00
	Greater Sydney	214.50 (± 67.90)	39.00	435.00
	Hunter	527.17 (± 363.84)	9.00	2,636.00
	Murray	458.51 (± 195.47)	129.00	1,081.00
	Northern Tablelands	870.56 (± 774.07)	26.00	5,441.00
	North Coast	726.53 (± 645.43)	18.00	4,184.00
	North West	1,529.68 (± 11,75.27)	28.00	7,479.00
	Riverina	2,996.26 (± 1,223.57)	269.00	7,343.00
	South East	544.84 (± 680.20)	23.00	6,221.00
**Year**	2021	748.88 (± 759.90)	8.00	5,713.00
	2022	815.27 (± 848.44)	3.00	7,557.00
	2023	1,009.94 (± 1,123.21)	5.00	7,518.00
	2024	1,181.78 (± 1,242.13)	8.00	10,771.00
**Month**	January	689.10 (± 760.72)	10.00	5,228.00
	February	983.10 (± 1,002.13)	8.00	7,343.00
	March	1,060.16 (± 1,129.08)	5.00	7,518.00
	April	1,182.65 (± 1,335.33)	15.00	10,771.00
	May	1,032.74 (± 999.26)	16.00	6,038.00
	June	871.20 (± 872.87)	8.00	5,935.00
	July	817.0 (± 9,782.11)	5.00	5,041.00
	August	792.45 (± 876.14)	8.00	5,376.00
	September	916.78 (± 1,074.46)	3.00	7,138.00
	October	881.25 (± 1,006.93)	12.00	7,138.00
	November	970.93 (± 1,054.94)	5.00	5,613.00
	December	964.02 (± 1,158.24)	9.00	7,301.00
**Season**	Autumn	1,089.25 (± 1,158.32)	5.00	10,771.00
	Spring	923.70 (± 1,045.85)	3.00	7,138.00
	Summer	884.70 (± 984.49)	8.00	7,343.00
	Winter	827.45 (± 846.54)	5.00	5,935.00
**Size of saleyard**	Small	132.06 (± 67.72)	3.00	258.00
	Medium	429.35 (± 101.69)	259.00	614.00
	Large	871.09 (± 167.26)	615.00	1,205.00
	Very Large	2,318.14 (± 1,157.81)	1,206.00	1,0771.00
**Saleyard location***	1	315.12 (± 465.99)	23.00	1,827.00
	2	18.23 (± 12.09)	3.00	46.00

	3	988.50 (± 816.99)	41.00	4,183.00
	4	394.70 (± 138.70)	213.00	632.00
	5	699.29 (± 605.52)	110.00	2,705.00
	6	214.50 (± 67.90)	39.00	435.00
	7	1,950.97 (± 1,413.19)	30.00	10,771.00
	8	1,319.07 (± 701.02)	38.00	4,184.00
	9	984.02 (± 1,345.47)	43.00	5,116.00
	10	1,065.93 (± 354.03)	521.00	2,004.00
	11	71.17 (± 42.45)	9.00	206.00
	12	298.25 (± 265.46)	46.00	1,223.00
	13	2,398.56 (± 1,603.83)	63.00	7,138.00
	14	606.32 (± 404.24)	42.00	2,682.00
	15	198.78 (± 99.72)	101.00	497.00
	16	458.51 (± 195.47)	129.00	1,081.00
	17	930.97 (± 446.73)	46.00	2,313.00
	18	1,789.89 (± 1,321.09)	52.00	3,255.00
	19	679.55 (± 498.31)	43.00	2,374.00
	20	688.94 (± 625.18)	35.00	3,141.00
	21	2,088.40 (± 807.93)	784.00	3,011.00
	22	1,561.07 (± 864.14)	42.00	3,744.00
	23	975.88 (± 704.97)	28.00	5,441.00
	24	701.26 (± 563.13)	29.00	3,729.00
	25	568.50 (± 446.52)	38.00	2,029.00
	26	430.24 (± 216.58)	27.00	1,453.00
	27	553.07 (± 299.55)	44.00	1,618.00
	28	331.54 (± 124.04)	142.00	574.00
	29	795.87 (± 498.09)	43.00	3,832.00
	30	642.96 (± 351.49)	27.00	1,543.00
	31	322.32 (± 178.38)	84.00	793.00
	32	440.36 (± 169.30)	87.00	794.00
	33	346.43 (± 125.96)	151.00	634.00
	34	130.19 (± 89.90)	30.00	402.00
	35	632.98 (± 342.36)	31.00	2,636.00
	36	591.94 (± 326.37)	26.00	2,379.00
	37	1,759.55 (± 1,296.26)	28.00	7,479.00
	38	168.22 (± 70.39)	35.00	410.00
	39	442.16 (± 635.98)	26.00	3,522.00
	40	1,795.40 (± 301.15)	1,489.00	2,230.00
	41	3,052.05 (± 1,223.74)	269.00	7,343.00
	42	232.10 (± 183.92)	18.00	1,293.00
	43	78.67 (± 31.05)	36.00	126.00
	44	674.37 (± 809.85)	32.00	6,221.00

*Saleyard locations have been coded for privacy

Table S5. Descriptive statistics of the number of sheep heads registered in the National Livestock Identification System (NLIS) from 2021 to 2024 at 4 saleyards located in NSW (Australia), stratified by region, year, month, season, size of saleyard and saleyard location.

Variable	Category	Number of sheep at each sale		
**Region**		**Mean (± SD)**	**Minimum**	**Maximum**
	Murray	11,964.72 (± 3,292.33)	4,151	21,674
	Riverina	27,853.37 (± 20,983.64)	1,053	79,986
	South East	11,665.15 (± 5,224.77)	1,700	32,194
**Year**	2021	18,146.81 (± 14,080.61)	1,053	56,214
	2022	17,571.61 (± 15,664.87)	2,065	75,055
	2023	19,731.25 (± 17,615.41)	2,392	79,986
	2024	23,064.55 (± 19,680.75)	2,954	71,771
**Month**	January	15,891.08 (± 13,891.80)	2,392	65,257
	February	19,965.12 (± 17,195.36)	4,151	71,771
	March	17,308.73 (± 14,613.68)	1,700	56,847
	April	18,104.54 (± 15,594.33)	3,786	62,654
	May	19,100.27 (± 16,167.67)	5,420	65,548
	June	19,232.72 (± 16,625.00)	2,783	63,408
	July	19,409.14 (± 17,829.94)	3,590	66,049
	August	19,451.01 (± 18,097.13)	4,558	57,913
	September	20,893.48 (± 18,192.44)	4,972	67,184
	October	23,504.25 (± 19,416.00)	3,729	68,027
	November	21,085.51 (± 17,296.36)	1,053	75,055
	December	22,122.63 (± 18,386.27)	2,065	79,986
**Season**	Autumn	18,176.68 (± 15,400.22)	1,700	65,548
	Spring	21,810.24 (± 18,249.74)	1,053	75,055
	Summer	18,976.39 (± 16,461.08)	2,065	79,986
	Winter	19,368.37 (± 17,470.24)	2,783	66,049
**Saleyard location***	1	11,964.72 (± 10,53.00)	79,986	21,674
	2	8,514.72 (± 3,444.39)	1,053	21,965
	3	46,693.60 (± 11,701.22)	6,994	79,986
	4	11,665.15 (± 5,224.77)	1,700	32,194
**Size of saleyard**	Small	6,427.55 (± 1,600.36)	1,053	8,602
	Medium	10,288.22 (± 968.47)	8,617	12,061
	Large	15,085.18 (± 2,788.06)	12,069	24,818
	Very Large	46,786.21 (± 11,170.19)	25,359	79,986

*Saleyard locations have been coded for privacy

Table S6. Descriptive statistics of the sale mortality rate of cattle registered in the National Livestock Identification System (NLIS) from 2021 to 2024 at 44 saleyards located in NSW (Australia), categorised by the factor saleyard location.

Saleyard location*	Sale Mortality Rate (%)		
	Mean(± SD)	Minimum	Maximum
1	0.00 (± 0.00)	0.00	0.00
2	0.00 (± 0.00)	0.00	0.00
3	0.023 (± 0.096)	0.00	0.748
4	0.00 (± 0.00)	0.00	0.00
5	0.00 (± 0.00)	0.00	0.00
6	0.00 (± 0.00)	0.00	0.00
7	0.019 (± 0.042)	0.00	0.328
8	0.043 (± 0.078)	0.00	0.561
9	0.013 (± 0.064)	0.00	0.389
10	0.011 (± 0.04)	0.00	0.231
11	0.00 (± 0.00)	0.00	0.00
12	0.00 (± 0.00)	0.00	0.00
13	0.048 (± 0.127)	0.00	1.140
14	0.00 (± 0.00)	0.00	0.00
15	0.00 (± 0.00)	0.00	0.00
16	0.015 (± 0.052)	0.00	0.274
17	0.036 (± 0.070)	0.00	0.387
18	0.007 (± 0.020)	0.00	0.062
19	0.00 (± 0.00)	0.00	0.00
20	0.006 (± 0.043)	0.00	0.524
21	0.00 (± 0.00)	0.00	0.00
22	0.004 (± 0.016)	0.00	0.099
23	0.025 (± 0.094)	0.00	0.944
24	0.015 (± 0.220)	0.00	3.174
25	0.00 (± 0.00)	0.00	0.00
26	0.00 (± 0.00)	0.00	0.00
27	0.003 (± 0.144)	0.00	2.040
28	0.00 (± 0.00)	0.00	0.00
29	0.003 (± 0.021)	0.00	0.180
30	0.00 (± 0.00)	0.00	0.00
31	0.00 (± 0.00)	0.00	0.00
32	0.00 (± 0.00)	0.00	0.00
33	0.00 (± 0.00)	0.00	0.00
34	0.00 (± 0.00)	0.00	0.00
35	0.00 (± 0.00)	0.00	0.00
36	0.017 (± 0.146)	0.00	2.079
37	0.003 (± 0.015)	0.00	0.152
38	0.00 (± 0.00)	0.00	0.00
39	0.00 (± 0.00)	0.00	0.00
40	0.00 (± 0.00)	0.00	0.00
41	0.077 (± 0.071)	0.00	0.433
42	0.043 (± 0.454)	0.00	4.762

43	0.00 (± 0.00)	0.00	0.00
44	0.024 (± 0.095)	0.00	1.250

*Saleyard locations have been coded for privacy

Table S7. Univariable linear regression model assessing the association between cattle sale mortality rate and saleyard location.

Variable	Estimate (± SE)	(95% CI)	Wald test *P*–value
**Saleyard location***			**< 0.001**
1	Ref.		
2	0.000 (± 0.005)	(–0.009, 0.009)	1
3	0.010 (± 0.003)	(0.004, 0.015)	0.001
4	0.000 (± 0.008)	(–0.016, 0.016)	1
5	0.000 (± 0.004)	(–0.008, 0.008)	1
6	0.000 (± 0.002)	(–0.005, 0.005)	1
7	0.017 (± 0.002)	(0.012, 0.021)	< 0.001
8	0.027 (± 0.002)	(0.023, 0.031)	< 0.001
9	0.000 (± 0.004)	(–0.008, 0.008)	1
10	0.006 (± 0.004)	(–0.002, 0.014)	0.153
11	0.000 (± 0.004)	(–0.008, 0.008)	1
12	0.000 (± 0.003)	(–0.007, 0.007)	1
13	0.022 (± 0.002)	(0.018, 0.026)	< 0.001
14	0.000 (± 0.003)	(–0.005, 0.005)	1
15	0.000 (± 0.005)	(–0.009, 0.009)	1
16	0.005 (± 0.002)	(–0.001, 0.011)	0.105
17	0.024 (± 0.002)	(0.019, 0.028)	< 0.001
18	0.007 (± 0.009)	(–0.010, 0.024)	0.420
19	0.000 (± 0.003)	(–0.005, 0.005)	1
20	0.003 (± 0.002)	(–0.002, 0.007)	0.232
21	0.000 (± 0.011)	(–0.022, 0.022)	1
22	0.004 (± 0.002)	(–0.001, 0.008)	0.133
23	0.018 (± 0.002)	(0.014, 0.022)	< 0.001
24	0.000 (± 0.002)	(–0.005, 0.005)	1
25	0.000 (± 0.002)	(–0.005, 0.005)	1
26	0.000 (± 0.003)	(–0.005, 0.005)	1
27	0.003 (± 0.002)	(–0.001, 0.008)	0.146
28	0.000 (± 0.007)	(–0.014, 0.014)	1
29	0.003 (± 0.002)	(–0.001, 0.008)	0.173
30	0.000 (± 0.003)	(–0.005, 0.005)	1
31	0.000 (± 0.003)	(–0.006, 0.006)	1
32	0.000 (± 0.004)	(–0.008, 0.008)	1
33	0.000 (± 0.006)	(–0.011, 0.011)	1
34	0.000 (± 0.003)	(–0.005, 0.005)	1
35	0.000 (± 0.002)	(–0.004, 0.004)	1
36	0.001 (± 0.002)	(–0.003, 0.006)	0.603
37	0.003 (± 0.002)	(–0.001, 0.007)	0.149
38	0.000 (± 0.003)	(–0.006, 0.006)	1
39	0.000 (± 0.003)	(–0.005, 0.005)	1
40	0.000 (± 0.011)	(–0.022, 0.022)	1

41	0.064 (± 0.002)	(0.060, 0.069)	< 0.001
42	0.000 (± 0.003)	(–0.006, 0.006)	1
43	0.000 (± 0.010)	(–0.020, 0.020)	1
44	0.009 (± 0.002)	(0.005, 0.014)	< 0.001

P-values in bold refer to the statistical significance or trend towards the significance of the predictive variable in the model; the significance of a category against the reference is reported in regular font

CI = confidence interval; Ref. = reference category; SE = standard error

*Saleyard locations have been coded for privacy

Table S8. Descriptive statistics of the sale mortality rate of sheep registered in National Livestock Identification System (NLIS) from 2021 to 2024 at 4 saleyards located in NSW (Australia), categorised by the factor saleyard location.

**Saleyard location***	**Sale Mortality Rate (%)**		
	**Mean (± SD)**	**Minimum**	**Maximum**
1	0.124 (± 0.053)	0.011	0.276
2	0.099 (± 0.053)	0.000	0.279
3	0.104 (± 0.996)	0.086	1.146
4	0.059 (± 0.040)	0.000	0.212

*Saleyard locations have been coded for privacy

Table S9. Univariable and Multivariable linear regression results for the outcome variable sheep sale mortality rate.

Variable	Estimate (± SE)	(95% CI)	*P*-value
**Univariable linear regression models**			
**Minimum daily temperature**	–0.001 (± 0.000)	(–0.001, –0.000)	**0.004**
**Maximum daily temperature**	–0.000 (± 0.000)	(–0.001, 0.000)	0.604
**Year**			0.212
2021	Ref.		
2022	–0.000 (± 0.005)	(–0.009,0.009)	0.982
2023	0.008 (± 0.005)	(–0.001, 0.017)	0.101
2024	0.006 (± 0.005)	(–0.003, 0.015)	0.182
**Month**			0.156
January	Ref.		
February	–0.003 (± 0.008)	(–0.019, 0.012)	0.697
March	0.001 (± 0.008)	(–0.014, 0.017)	0.859
April	0.000 (± 0.008)	(–0.016, 0.017)	0.975
May	0.007 (± 0.008)	(–0.009, 0.022)	0.387
June	0.009 (± 0.008)	(–0.006, 0.025)	0.237
July	0.011 (± 0.008)	(–0.005, 0.026)	0.185
August	0.014 (± 0.008)	(–0.002, 0.029)	0.079
September	0.009 (± 0.008)	(–0.007, 0.024)	0.264
October	0.006 (± 0.008)	(–0.009, 0.022)	0.419
November	0.015 (± 0.008)	(–0.001, 0.030)	0.063
December	0.022 (± 0.009)	(0.004, 0.040)	0.016
**Season**			0.174
Autumn	Ref.		
Spring	0.007 (± 0.005)	(–0.002, 0.094)	0.127
Summer	0.001 (± 0.005)	(–0.008, 0.010)	0.852
Winter	0.008 (± 0.005)	(–0.001, 0.017)	0.069
**Size of saleyard**			0.978
Small	Ref.		
Medium	0.001 (± 0.005)	(–0.008, 0.010)	0.829
Large	0.000 (± 0.005)	(–0.009, 0.009)	0.961
Very large	0.002 (± 0.005)	(–0.007, 0.011)	0.691
**Region**			**< 0.001**
South East	Ref.		
Murray	0.055 (± 0.004)	(0.046, 0.063)	< 0.001
Riverina	0.033 (± 0.004)	(0.026, 0.040)	< 0.001
**Saleyard location**			**< 0.001**
1	Ref.		
2	0.055 (± 0.004)	(0.046, 0.063)	< 0.001
3	0.036 (± 0.004)	(0.028, 0.044)	< 0.001
4	0.030 (± 0.004)	(0.022, 0.038)	< 0.001
**Multivariable linear regression model**			

**Minimum daily temperature**	–0.0004 ± 0.000	(–0.001, 0.000)	**0.093**
**Saleyard location***			**< 0.001**
1	Ref.		
2	0.053 (± 0.004)	(0.044, 0.061)	< 0.001
3	0.035 (± 0.004)	(0.026, 0.043)	< 0.001
4	0.029 (± 0.004)	(0.020, 0.037)	< 0.001

P-values in bold refer to the statistical significance of the predictive variable in the model; the significance of a category in comparison with the reference value is reported in regular font

CI = confidence interval; Ref. = reference category; SE = standard error

*Saleyard locations have been coded for privacy
